# VACTERL Association in a Fetus With a Normal Genetic Profile

**DOI:** 10.7759/cureus.65809

**Published:** 2024-07-30

**Authors:** Sneha Jawalkar, Aarushi Goswami, Neelamma Patil, Savitri Nerune

**Affiliations:** 1 Pathology and Laboratory Medicine, Shri B.M. Patil Medical College, Hospital & Research Centre, BLDE (Deemed to be University), Vijayapura, IND; 2 Obstetrics and Gynaecology, Shri B.M. Patil Medical College, Hospital & Research Centre, BLDE (Deemed to be University), Vijayapura, IND

**Keywords:** talipes equinovalgus, talipes equinovarus, club foot, hemivertebrae, tetralogy of fallot

## Abstract

VACTERL association is a statistical association of vertebral(V), anal(A), cardiac(C), tracheoesophageal(TE), renal(R), and limb(L) abnormalities (VACTERL). Diagnosis of VACTERL can be made if anomalies in three or more organ systems indicated by the acronym are present and no other recognizable pattern of human malformations exists. We hereby present a case of a primigravida in her 20s, whose prenatal scan at 13 weeks of gestation revealed a fetus having a short spine, single outflow tract, and increased nuchal translucency. Chromosome microarray on amniotic fluid showed no quantitative genomic imbalance. A repeat scan at 21 weeks disclosed lumbar scoliosis with hemivertebrae, tetralogy of Fallot, and a single umbilical artery. After undergoing counselling, in light of the adverse outcomes and poor quality of life of the offspring, the parents decided to terminate the pregnancy. The abortus was subsequently sent to the histopathology lab for autopsy. The gross examination of the fetus unveiled an umbilical cord with only two vessels, right congenital talipes equinovarus (club foot), left congenital talipes equinovalgus, and right foot polydactyly. Internal examination of the cardiovascular system verified the presence of tetralogy of Fallot. The kidogram of the fetus was consistent with a butterfly vertebral body of D11 and D12 with right-side hemivertebrae. This case illustrates the importance of foetal autopsy for confirming the prenatal diagnosis and identifying abnormalities in syndromes or associations. The classification especially plays a crucial role in the case of recurrent abortions to point out the underlying aetiology and counsel the parents regarding the recurrence risk.

## Introduction

VACTERL association is an acronym that includes a comprehensive spectrum of congenital anomalies that can involve Vertebral, Anorectal, Cardiac, Tracheo-Esophageal, Renal, and Limb anomalies [1,2,3]. For diagnosis of VACTERL association, at least three out of the above-mentioned anomalies should be present, in the absence of other phenotypic or genetic features indicative of an alternative diagnosis [1,2,4]. The prevalence of this association is calculated to be one in 10,000 to one in 40,000 in newborns [1,2,4]. There is strong clinical and genetic evidence for casual heterogenicity in patients with VACTERL association. Therefore, a vast majority of cases have been reported in isolated individuals and families [5]. With respect to VACTERL association, we should be mindful of the fact that it is common for a patient to display a completely normal genetic profile but still be affected by the association as it can be an accessory to various maternal risk factors [5]. 

## Case presentation

A primigravida in her late 20s presented for a routine prenatal check-up. Her first-trimester radiological scan showed various abnormal findings including reduced foetal movement, short spine, and single outflow tract. There was no history of consanguinity, diabetes, or smoking, and a history of exposure to organic chemicals or pesticides was stated to be negative. She also reported no previous history of congenital or genetic disorders in immediate family members and no complaints of fever or any other infection. Table 1 reflects on all relevant investigations she underwent.

**Table 1:** Investigations

S. No.	Investigation	Result
1.	Antenatal Scan at 13 weeks	Reduced foetal movement, short spine, and single outflow tract.
2.	Chromosome Microarray	No quantitative genomic imbalance
3.	Penta screen test -includes serum alpha-fetoprotein levels, free beta human chorionic gonadotropin, inhibin, pregnancy-associated plasma protein-a, and placental growth factor	Increased risk for pre-eclampsia for more than 37 weeks of gestation. Calculated risk for trisomy 21, 18, 13, and neural tube defects was very low. (As it was a precious pregnancy the couple decided to continue with the pregnancy)
4.	Antenatal Scan at 21 Weeks	Lumbar scoliosis with hemivertebrae, single umbilical artery, and tetralogy of Fallot.
5.	Post 21 weeks antenatal scans	The couple was counselled about the outcome of the pregnancy and poor quality of life of the offspring, following which they decided to terminate the pregnancy at 21 weeks. The fetus was sent for autopsy examination.
6.	Fetal Autopsy showed limb anomaly, cardiac anomaly, & vertebral anomaly fulfilling the criteria for VACTERL association.	External examination showed- Bilateral Club foot (Asymmetrical – right congenital talipes equinovarus and left congenital talipes equinovalgus) and polydactyly of the right foot. (Figure 1) On further examination, the cut surface of the umbilical cord showed the presence of 2 vessels. The remainder of the external examination was normal. Heart displayed normal localization with abnormal findings. It measured 2x1x0.5 cm and weighed 7 gm, showing overriding of the aorta originating more from the right ventricle and less from the left ventricle, ventricular septal defect, and absence of pulmonary trunk (Figure 2). The right ventricular wall was thickened (Figure 2) measuring 4 mm (normal thickness 2 mm) and the left ventricular wall measuring 3 mm (normal thickness 2 mm). These findings confirmed tetralogy of Fallot. All other cardiovascular system findings and measurements were within normal range. On microscopy section studied from the umbilical cord showed the presence of two vessels. The thymus, bilateral lungs, spleen, liver, bilateral kidneys, and adrenals showed normal histology.
7.	X-ray findings of the fetus (Figure 3)	D11 and D12 butterfly anomaly (hemivertebrae), segmental anomaly of all lumbar vertebrae with widening and splaying of anterior elements.

**Figure 1:**
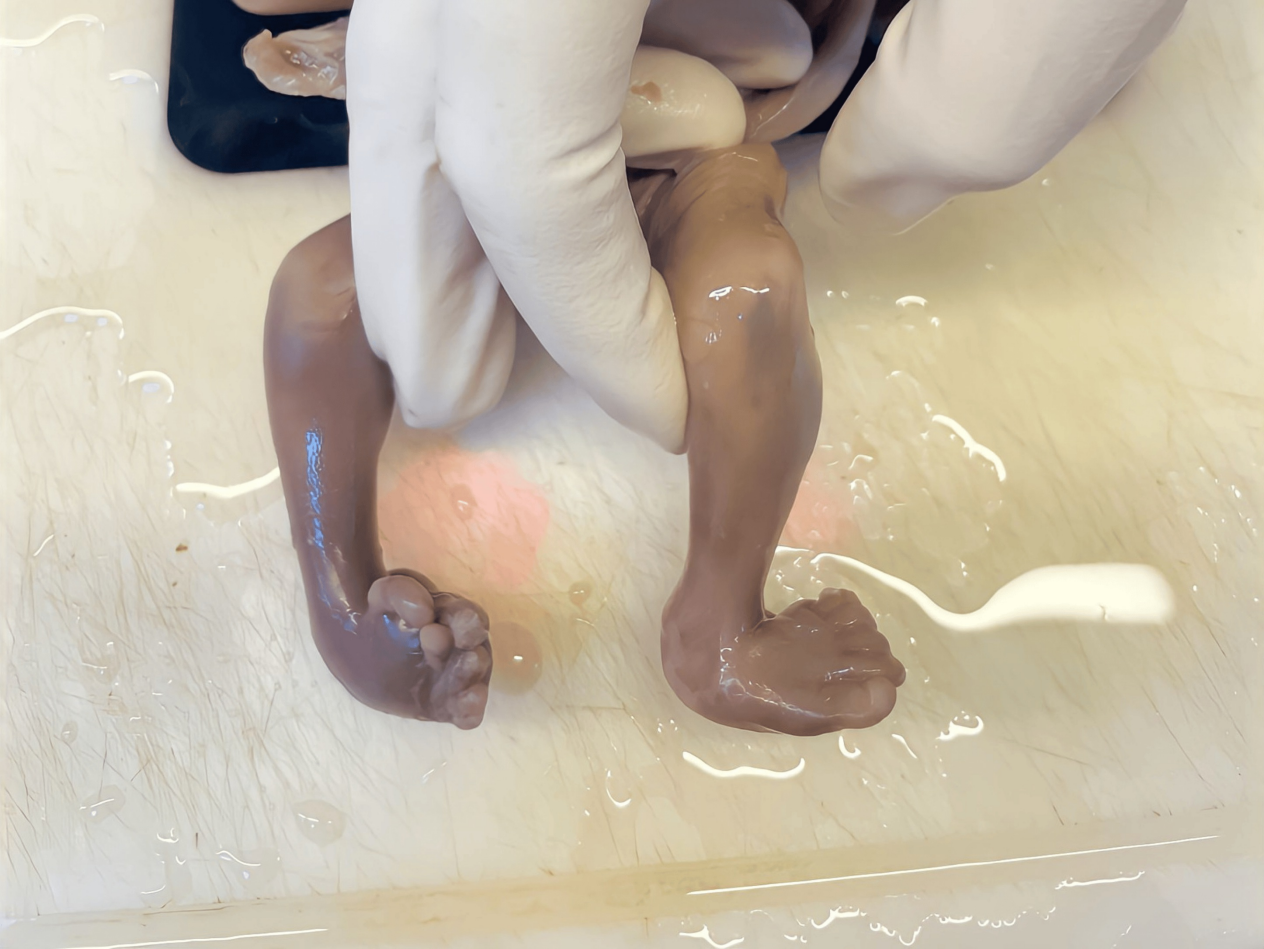
Fetal autopsy showing bilateral club foot (asymmetrical - right congenital talipes equinovarus and left congenital talipes equinovalgus) and polydactyly of the right foot

**Figure 2:**
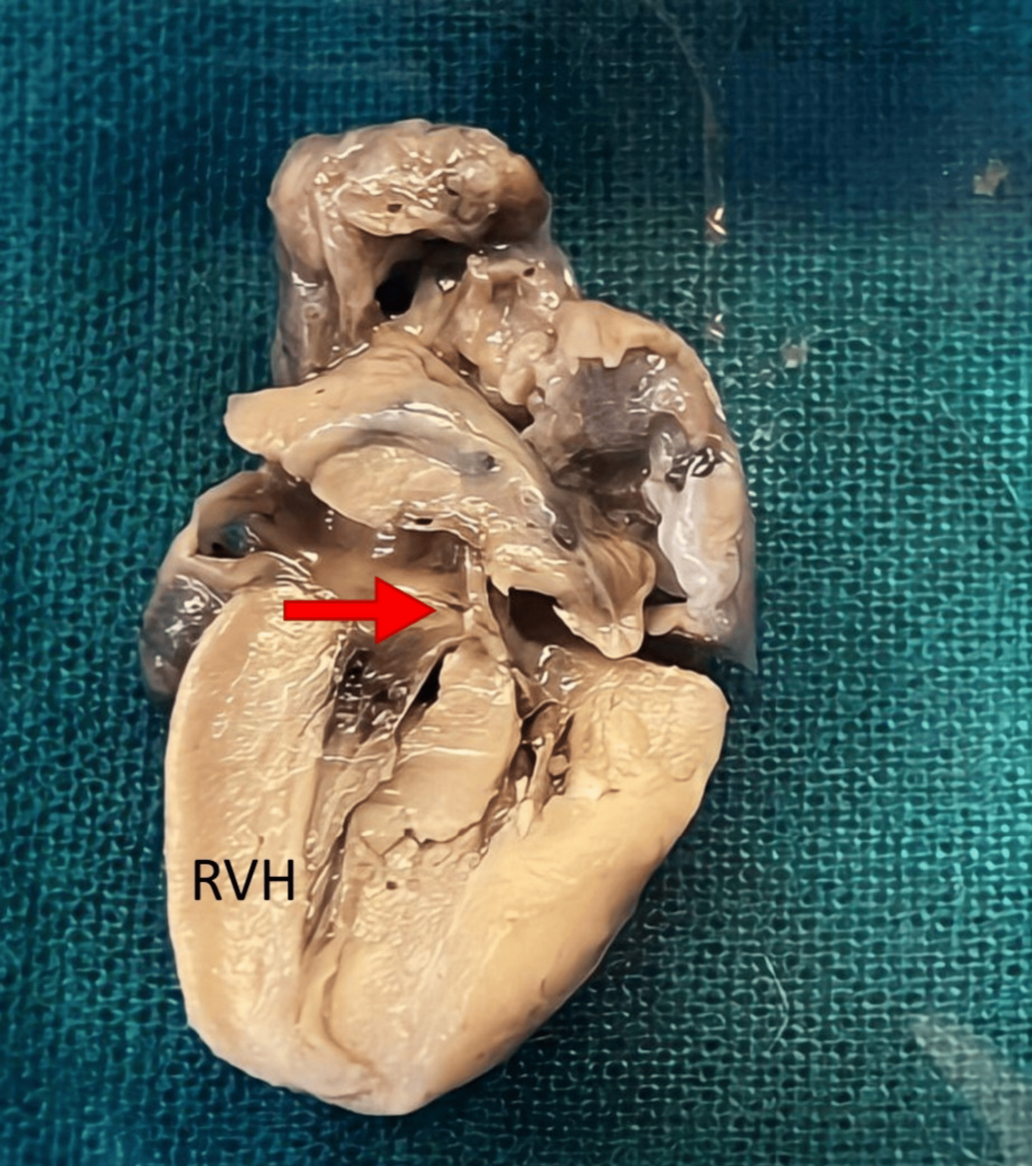
fetal autopsy gross picture of the longitudinal cut section of heart showing ventricular septal defect and overriding of the aorta (red arrow) along with right ventricular hypertrophy (RVH).

**Figure 3:**
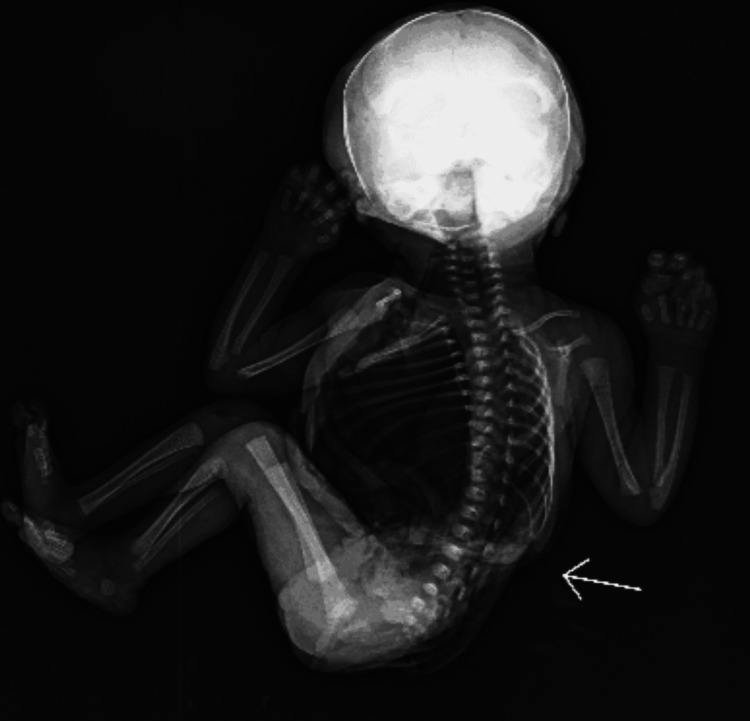
X-ray findings of the abortus showing D11 and D12 butterfly anomaly (hemivertebrae)(white arrow), segmental anomaly of all lumbar vertebrae with widening and splaying of anterior elements.

All other gross and histopathological findings appeared to be normal.

## Discussion

A random co-occurrence of the structural malformations named VATER (Vertebral defects, Anal atresia, T-E fistula with esophageal atresia, Radial and Renal dysplasia) association was first described by Quan L and Smith D in the year 1973 [6]. Later, Cardiac(C), Limb (L), and Single umbilical artery(S) were included and the terms VACTERL/VACTERLS were coined by Temtamy and Miller [7]. Lately, hydrocephalus was added by Zen et al. and the term VACTERL-H was described [8]. Table 2 elaborates on the anomalies in relation to VACTERL association.

**Table 2:** Detailed description of anomalies

Anomaly	Incidence	Most common deformities associated
Vertebral	60-80%	Segmentation defects(hemivertebrae, butterfly vertebrae, wedge vertebrae), vertebral fusion, supernumerary or absence of vertebrae, vertebral dysplasia
Anal anomalies	55-90%	Imperforate anus, anal atresia
Cardiac anomaly	40-80%	Structural heart anomalies, Ventricular Septal Defect, Atrial Septal Defect, and tetralogy of Fallot
Tracheoesophageal anomaly	50-80%	Tracheo-esophageal fistula. May be associated with pulmonary anomalies as these may share a common structural anatomical cause with Tracheoesophageal fistula
Renal anomalies	50-80%	Unilateral renal agenesis, horseshoe kidney, and cystic and dysplastic kidney.
Limb anomalies	40-50%	Radial anomalies, including thumb aplasia/ hypoplasia, polydactyly and lower limb anomalies

Out of the above commonly occurring defects, our case showed vertebral defects (butterfly vertebrae), cardiac anomaly (tetralogy of Fallot), limb defects (polydactyly and talipes equinovalgus and equinovarus), and single umbilical artery.

The aetiology of VACTERL association remains unknown and can occur sporadically in many cases. Some suggest that it may be due to development defects during blastogenesis (two to four weeks of gestation) due to disruption of the sonic hedgehog (SHH) signal transduction pathway that compromises the development of multiple organs at the same instant [4].

One of the major maternal risk factors associated with the occurrence of VACTERL association include assisted reproductive technique. Other risk factors can be listed as primiparity, pregestational obesity, inconsistency in folic acid supplementation, and continuous smoking from a period of three to 10 weeks after conception [9].

Other than maternal risk factors, in a study conducted by Solomon BD et al, it was highlighted that a small fraction of patients with VACTERL association can be an isolated individual or families as seen in our case. Other reported causes include mitochondrial dysfunction, pathogenic copy number variations, heterozygous mutation in HODX3, and heterozygous/ hemizygous mutation in ZIC 3 [5].

According to a study conducted by Miller OF et al, in children born with VACTERL association, approximately one-quarter of malformations of VACTERL association were not made until adulthood; this may be due to the lack of awareness of the association and related conditions [1,2]. These late-diagnosed cases lead to medically significant issues later in life, leading to many debilitating anomalies that compromise the quality of life (Table 3) [5].

**Table 3:** A detailed description of the sequelae of malformations and long-term outcomes associated with VACTERL association. Based on Solomon [5]. UTI: urinary tract infection; GE: Gastroesophageal; VACTERL: vertebral(V), anal(A), cardiac(C), tracheoesophageal (TE), renal(R) and limb(L) abnormalities

Features	Early potential medical complication	Late (post-infant ) medical complication
Vertebral anomalies	Scoliosis, tethered cord, syrinx	Progressive scoliosis, back pain, osteoarthritis, tethered cord, syrinx
Anal atresia	Obstruction	Incontinence, constipation, immobility, sexual dysfunction,
Cardiac malformation	Compromised cardiopulomonary function, dysrhythmias	Compromised cardiac function, dysrhythmias
Tracheoesophageal fistula	Inability to feed, respiratory compromise, pneumonia	GE reflux, increased risk of gastroesophageal cancers, reactive airway disease,
Renal anomalies	Vesicouretral reflux, hydronephrosis, UTI	UTI, nephrolithiasis, impaired renal function
Limb anomalies	Functional impairment	Functional impairment

It is noteworthy that there is very limited literature discussing the incidence of asymmetric club foot in VACTERL association due to its rarity and variability in presentation, making it a one-of-a-kind case.

Due to a large array of defects and anomalies with no specific genetic defect, VACTERL association has become a diagnosis on the basis of exclusion and results in a panoramic list of differentials that encompasses Alagille syndrome most commonly that was excluded in this case due to the absence of typical facial and ophthalmic anomalies, arteriohepatic dysplasia, intrahepatic bile duct paucity, pulmonic valvular and peripheral arterial [10].

Other differential diagnoses due to multiple overlying features with VACTERL association comprise 22q11.2 deletion syndrome, Fanconi anaemia, Feingold syndrome, Holt-Oram syndrome, oculo-auriculo-vertebral syndrome, and Towes-Brocks syndrome [5].

It is important to keep in mind that the evidence of a single umbilical artery in many cases acts as the first indication of a diagnosis of VACTERL Association [3].

With respect to VACTERL association, Miller OF et al observed a neonatal mortality of 28%, whereas Kolon T.F. reported a 100% survival rate with a follow-up of 5-7 years [11].

## Conclusions

Through this case report, we learn that a spectrum of structural foetal malformations can occur with an absolutely normal genetic profile attributing to various other preventable maternal risk factors. Foetal autopsy is vitally important for confirming the prenatal diagnosis, recognising additional malformations, and providing an association with genetic syndromes. 

Prompt counselling of parents and family members is of utmost importance so as to decide the further course of pregnancy or consider termination of pregnancy to reduce the socioeconomic burden on the family as well as society.
